# Changes in Oxidative Status Biomarkers in Saliva and Serum in the Equine Gastric Ulcer Syndrome and Colic of Intestinal Aetiology: A Pilot Study

**DOI:** 10.3390/ani12050667

**Published:** 2022-03-07

**Authors:** María Dolores Contreras-Aguilar, Camila Peres Rubio, Luis Guillermo González-Arostegui, María Martín-Cuervo, Jose J. Cerón, Ignacio Ayala, Ida-Marie Holm Henriksen, Stine Jacobsen, Sanni Hansen

**Affiliations:** 1 Interdisciplinary Laboratory of Clinical Analysis of the University of Murcia (Interlab-UMU), Department of Animal Medicine and Surgery, Veterinary School, Regional Campus of International Excellence Mare Nostrum, University of Murcia, Campus de Espinardo, 30100 Murcia, Spain; camila.peres@uab.cat (C.P.R.); luisgarostegui@gmail.com (L.G.G.-A.); jjceron@um.es (J.J.C.); 2 Department of Animal and Food Science, School of Veterinary Science, Universitat Autònoma de Barcelona, 08193 Cerdanyola del Vallès, Spain; 3 Medicine Animal, Faculty of Veterinary Medicine of Cáceres, University of Extremadura, Avenida de la Universidad S-N, 10002 Cáceres, Spain; mmcvet@hotmail.com; 4 Department of Animal Medicine & Surgery, Veterinary School, Regional Campus of International Excellence Mare Nostrum, University of Murcia, Campus de Espinardo, 30100 Murcia, Spain; iayape@um.es; 5 Department of Veterinary Clinical Sciences, Section Medicine and Surgery, University of Copenhagen, Agrovej 8, DK-2630 Taastrup, Denmark; ida-marie.boll@sund.ku.dk (I.-M.H.H.); stj@sund.ku.dk (S.J.); sannih@sund.ku.dk (S.H.)

**Keywords:** horse, EGUS, colic, redox profile, saliva

## Abstract

**Simple Summary:**

Gastric ulcers and colic of intestinal aetiology (CIE) are highly prevalent diseases in domestic horses, with a negative impact on equine sport and breeding life. Therefore, the studies investigating possible biomarkers for their diagnosis or clarifying their pathophysiology are of high interest. Oxidative status changes have been reported in both diseases, but only in the blood. However, saliva may be a relevant source of oxidative status biomarkers not yet assessed in horses with interesting advantages, owing to its non-invasive collection. Hence, this study aimed to validate in both saliva and serum automated assays for the measurement of oxidative status biomarkers and assess them in horses suffering gastric ulcer diseases (squamous and/or glandular) and CIE, studying their possible relationship with their inflammatory and immunity status. It was found that horses with glandular mucosa ulcers showed higher levels of some antioxidant and oxidative biomarkers in saliva correlating with a marker of the immune system such as salivary adenosine deaminase. Horses suffering from CIE had increases in serum uric acid associated with their systemic inflammatory response and outcome of the disease. In conclusion, some oxidative status analytes can be automatically measured in horses’ saliva and serum and may potentially be assessed as biomarkers of gastric ulcers and CIE.

**Abstract:**

Changes in the oxidative status of the blood of horses suffering from gastric ulcers and colic of intestinal aetiology (CIE) have been reported. However, saliva can also be a source of biomarkers of oxidative status. Therefore, this study aims to validate automated assays for the measurement of oxidative status biomarkers (ferric reducing ability of saliva/serum—FRAS/FRAP, cupric reducing antioxidant capacity—CUPRAC, the Trolox equivalent antioxidant capacity—TEAC, uric acid, and advanced oxidation protein products—AOPP) in the saliva and serum of horses, to assess their changes in the different ulcer gastric diseases (squamous—ESGD and glandular—EGGD) and CIE, and to evaluate their relationship with serum amyloid A (SAA), adenosine deaminase (ADA), and the systemic inflammatory response syndrome (SIRS) status. The assays showed a low imprecision and good linearity with enough sensitivity in both fluids. In EGGD, higher levels of FRAS, uric acid, and AOPP in saliva were observed compared to the healthy group, correlating with the salivary ADA levels. Horses with CIE showed increases in uric acid concentrations in serum associated with their SIRS status and outcome of the disease. In conclusion, analytes related to the oxidative status can be measured in the saliva and serum from horses by automated assays, and some of them can potentially be assessed as biomarkers in horses with gastric ulcers and CIE.

## 1. Introduction

Blood is the sample most frequently used for biomarker assessment in human and veterinary medicine [1]. However, saliva can be used as a complement or even an alternative to blood to measure selected analytes, with the advantage of being easy to collect in a non-invasive way without any need for specialized training [2]. Some studies have measured various analytes in horse saliva by automated assays [3,4,5]. In addition, an oxidative stress profile in the saliva of other species such as pigs and sheep has been assessed [6,7]. However, oxidative stress in horse saliva has not previously been reported to the best of the authors’ knowledge, and, currently, there are no analytically validated assays described for the evaluation of oxidative stress in the saliva of horses.

Equine Gastric Ulcer Syndrome (EGUS) and colic of intestinal aetiology (CIE) are diseases with a significant impact in equine sport and breeding due to their prevalence and costly treatment [8,9]. In the case of EGUS, the European College of Equine Internal Medicine (ECEIM) Consensus Statement (2015) distinguishes two different diseases depending on which region anatomically is affected: the Equine Squamous Gastric Disease (ESGD) and the Equine Glandular Gastric Disease (EGGD) [10]. Their multifactorial nature and non-specific clinical signs have increased the interest in identifying possible analytes that could both help clarify the pathophysiology and serve as biomarkers of disease [9,11,12]. In this sense, oxidative changes in serum have been reported in horses with EGUS independent of the EGUS type [9]. However, the presumable differentiated pathogenesis process between ESGD and EGGD [10] makes it of interest to differently evaluate those biomarkers according to different entities’ diseases. On the other hand, the CIE is a major cause of death in horses [13], and there is a longstanding tradition for research into new biomarkers that could be used for evidence-based decisions regarding the treatment and management of colic cases [14,15]. In CIE, changes in oxidant/antioxidant biomarkers associated with leukocyte activation have been reported in serum from horses with large intestinal ischemia [16]. However, to the best author’s knowledge, there are no studies about changes in biomarkers of oxidative stress in saliva in the EGUS types or CIE.

Therefore, this study aims: (1) to validate assays for the measurement of selected biomarkers of oxidative status in saliva and, for comparative purposes, serum of horses; (2) to assess the possible differences in these analytes if suffering from EGSG, EGGD, or CIE; (3) to evaluate the possible relationship of these analytes in serum and saliva with the inflammation status assessed by the serum amyloid A (SAA) [5,17] or their systemic inflammatory response status (SIRS) [18], and with a biomarker of cell-mediated immunity such as adenosine deaminase (ADA) [4,19,20]. The biomarkers of oxidative status were selected based on their adaptability to automated analysers and included antioxidants biomarkers such as the total antioxidant capacity (TAC) evaluated by three different assays (the ferric reducing ability of saliva/serum (FRAS/FRAP), cupric reducing antioxidant capacity (CUPRAC), and the Trolox equivalent antioxidant capacity (TEAC)) and uric acid, and oxidant biomarkers such as the advanced oxidation protein products (AOPP).

## 2. Materials and Methods

### 2.1. Diseased and Healthy Populations

The horse population enrolled in this study originated from the Large Animal Teaching Hospital at the University of Copenhagen and the Veterinary Teaching Hospital of the University of Extremadura. Samples were collected from February 2020 to May 2021. The horses’ age, sex, and breed were recorded.

The diseased population were diagnosed at those hospitals by certified internists (M.M.-C., S.H.).

Horses suspected of EGUS (e.g., due to riding issues, weight loss, pain behaviours, or changes in temperament) [10] were referred to the hospital the day before the gastroscopy. In both centres, the same protocol for detecting the disease consisting of anamnesis, physical examination (including evaluation of heart rate (HR), respiratory rate (RR), rectal temperature, colour of mucous membranes, capillary refill time, and borborygmi), haematology, plasma biochemistry, and gastroscopic technique was performed. Horses fasted for 16 h before the gastroscopy, which was performed as previously described [9]. The horses were sedated with detomidine (0.01 mg/kg i.v; Domosedan, Orion Pharma Animal Health A/S, Copenhagen, Denmark), butorphanol tartrate (0.01 mg/kg i.v; Dolorex, MSD Animal Health, Copenhagen, Denmark), and/or acepromazine (0.03 mg/kg i.v; Pharmaxim AB, Helsingborg, Sweden). For the gastroscopic technique, a flexible video endoscope (3 m, dia. 9.8 mm; Karl Storz, Denmark) was used. Once the squamous and glandular mucosa from EGUS horses was documented, the gastroscopic pictures and videos were used for the EGUS diagnosis [21] and classified according to the ECEIM Consensus Statement from 2015 [10], whereby horses were stratified into ESGD, EGGD, or both (ESGD + EGGD).

Colic of intestinal aetiology was diagnosed in both centres based on history, physical examination (including evaluation of HR, RR, rectal temperature, colour of mucous membranes, capillary refill time, and borborygmi), haematology, plasma biochemistry, examination per rectum, transabdominal ultrasonography, gastric reflux, abdominocentesis results, exploratory laparotomy, and/or response to treatment. Whether each suffered a systemic inflammatory response syndrome (SIRS) was evaluated by the SIRS score (4 point-score) proposed by Roy et al. [18]. According to this, SIRS was graded based on the number of abnormal clinical variables; i.e., HR (>52 beats/min), RR (>20 breath /min), WBC count (outside the range of 5.0–12.5 × 10^9^/L), and rectal temperature (outside the range of 37.0–38.5 °C). When performed, necropsy diagnoses were recorded.

The healthy population was selected from privately owned horses admitted at the hospitals for routine health checks without any signs of illness to confirm their health status, in which a gastroscopy study, as previously described, was performed to discount EGUS (ESGD grading system equal to 0 meaning that epithelium was intact with no hyperkeratosis areas, nor any glandular lesions). At the hospital, they showed normal findings on a physical examination (HR, RR, rectal temperature, colour of mucous membranes, capillary refill time, borborygmi) and no haematological or biochemical abnormalities.

### 2.2. Sampling

The saliva was obtained as previously reported [3,22]. The sponges (Esponja Marina, La Griega E. Koronis, Madrid, Spain) soaked with saliva were placed in a commercially available device immediately after sampling (Salivette, Sarstedt, Aktiengesellschaft & Co, Nümbrecht, Germany). The cases were only included if guaranteed that horses did not receive any feed for at least 12 h and if the dirtiness degree of the saliva was 0 or 1 according to the colour scale previously reported (0–4 score) [23]. After saliva sampling, blood was obtained by jugular venepuncture and transferred into tubes containing a clot activator (Becton Dickinson Vacutainer Systems Europe). Tubes with saliva and blood were centrifuged at 3000× *g* for 10 min at 4 °C within 30 min of sampling. Saliva and serum were then transferred into Eppendorf tubes and stored at −80 °C until analysis. Horses that yielded serum samples with visual gross haemolysis or blood-contaminated saliva samples were excluded from the study.

The samplings in the EGUS and healthy population were performed before intravenous sedation and gastroscopy but immediately after the horses were placed in the examination stand. In the CIE group, the samples were obtained after the arrival at the hospital, immediately after horses were placed in the examination room.

### 2.3. Salivary and Plasma Biomarkers

The TAC analytes (FRAS/FRAP, CUPRAC, and TEAC) and the biomarker of oxidant status AOPP were measured by assays previously adapted to automated analysers and used for measurements in saliva and serum of different veterinary species such as pigs, sheep, or cows [7,24,25]. The ratio AOPP/albumin was calculated and reported since it has been described in humans and cows that AOPP is derived predominantly from serum albumin, and this ratio proved to be a more sensitive indicator of oxidative stress [26]. Albumin was evaluated using a commercial kit from Beckman (Albumin, Beckman Coulter Inc., Fullerton, CA, USA), previously validated in horse blood [3]. In saliva, this ratio was not assessed due to the almost undetectable albumin levels measured by the Beckman assay [3]. The antioxidant uric acid was evaluated by a commercially available spectrophotometric method (Uric Acid reagent OSR6698 Beckman Coulter AU analysers, Switzerland) according to the manufacturer’s instructions.

The ADA enzyme in saliva and serum (total ADA (ADAt), ADA isoenzyme 1 (ADA1), and isoenzyme 2 (ADA2)) was determined by a commercially available spectrophotometric automated assay (Adenosine Deaminase assay kit, Diazyme Laboratories, Poway, CA, USA) previously reported and validated in horses (lower limit of detection (LLOD) = 0.07 IU/L) [4]. ADA2 was not determined in serum since ADA2 levels are very low or absent in horses and, therefore, most serum ADAt is present as ADA1 [4]. SAA was measured by a commercially available immunoturbidometric automated assay (LZ SAA, EIKEN Chemical Co Ltd., Tokyo, Japan) developed for human serum but validated in horses (LLOD = 0.48 mg/L) [27]. Serum amyloid A was not determined in saliva since the immunoturbidimetric assay is not sensitive enough to detect SAA in saliva [3]. All the described assays above were carried out on an automated biochemical analyser (Olympus Diagnostica GmbH AU 400, Beckman Coulter, Ennis, Ireland).

### 2.4. Analytical Validation of the Oxidative Stress Profile

The salivary and serum analytical validation for the FRAS/FRAP, CUPRAC, TEAC, uric acid, and AOPP analytical assays was determined assessing the following characteristics:

Imprecision. Calculated as the intra-assay precision by the within-run coefficient of variation (CV, %). For this purpose, three pools of saliva and serum samples with high, medium, and low levels of each of the above analytes (Table 1) were used, and they were analysed five times in a single analytical run. The pools were obtained by mixing the same volume of saliva from two healthy or diseased horse samples for each analyte.

Accuracy. Indirectly estimated by linearity under dilution. It was calculated by serially diluting (1:2, 1:4, and 1:8) two pools of saliva/serum obtained as described above (Table 2) with deionized water. Then, linear regression between the observed and the expected results was performed, and the slope, y-intercept, and R^2^ were calculated.

Sensitivity. Determined by the LLOD and the lower limit of quantification (LLOQ). The LLOD is defined as the lowest values of the analyte that could be distinguished from a specimen of zero value. It was calculated as a mean value plus 3 standard deviations of 13 replicate determinations of the zero standard (deionized water). The LLOQ is the lowest level of the analyte that can be measured above the limit of detection with a CV < 20%, and it was calculated from a pool of saliva/serum obtained as above described serially diluted with deionized water (1:2, 1:4, 1:8, 1:10, 1:20) and each dilution was analysed in five replicates in the same run. CVs for each dilution were estimated for the intra-assay precision.

### 2.5. Data Analysis

Data were checked for normality using the Shapiro–Wilk normality test. Arithmetic means and 95% confidence interval (CI) if data showed normal distribution or medians and interquartile range (IQR) if not normally distributed, standard deviation (s.d.), CVs, and linear regression analysis for the linearity under dilution test were calculated using an Excel 2000 h spreadsheet and Graph Pad Software Inc. (San Diego, CA, USA).

The possible differences in the salivary and serum oxidative stress profile, ADAt in saliva and serum, and ADA isoenzymes in saliva between the healthy population and the horses suffering ESGD, EGGD, ESGD + EGGD, or CIE, and between the healthy population and those horses which survived CIE vs. those that did not (non-survivors), were evaluated by a one-way ANOVA test with Tukey’s adjustment for multiple comparisons or by the Kruskal–Wallis test followed by Dunn’s multiple comparisons test, depending on whether the data were normally or abnormally distributed, respectively. A stand-alone power program for statistical testing (G-Power) [28] was employed to perform a post hoc analysis and to determine if a power over 80% (β error ≤ 0.20) was achieved with the number of horses evaluated. Possible linear correlations between the salivary and serum redox profile with (1) inflammatory (SAA) and immunological (salivary and serum ADAt, and salivary ADA1 and ADA2) biomarkers; and (2) the SIRS score; were calculated by the Spearman correlation test (r). An r value from 0.90 to 1 was considered to have very high correlation; 0.70 to 0.90, high correlation; 0.50 to 0.70, moderate correlation; 0.30 to 0.50, low correlation; and less than 0.30, little if any correlation [29]. These statistical analyses were performed using Graph Pad Software Inc. (GraphPad Prism, version 9.1.0 for macOS; San Diego, CA, USA). Values of *p* ≤ 0.05 were selected to indicate significance in all analyses).

## 3. Results

### 3.1. Diseased and Healthy Populations

A total of 23 horses with EGUS and 22 horses with CIE were enrolled in this study. Details of ESGD (*n* = 6, average age = 11.0 ± 5.0 years), EGGD (*n* = 8, average age = 11.3 ± 2.1 years), ESGD+EGGD (*n* = 9, average age = 10.4 ± 3.7 years), CIE (*n* = 22, average age = 12.1 ± 4.8 years), and healthy populations (*n* = 14, average age of 11.5 ± 6.2 years) are provided in Supplementary Table S1. In the CIE group, 14 horses survived and 8 horses died or were euthanized.

### 3.2. Analytical Validation of the Oxidative Stress Biomarkers

The imprecision intra-assay of the oxidative stress biomarkers evaluated is shown in Table 1. Intra-assay CVs (%) for the redox profile in saliva and serum were <10% for the specimens with low, medium, and high levels evaluated. The linearity under dilution study in the redox profile in saliva and serum yielded an R^2^ > 0.960 for both pool specimens (Table 2). In both specimens, slopes were not significantly different from one, and intercepts were not significantly different from zero in all the redox biomarkers evaluated for both saliva and serum.

The LLOD and LLOQ of the oxidative status profile evaluated are shown in Table 3. No values lower than LLOQ were reported, with the exception of some values of: TEAC measured in saliva in the healthy population (*n* = 2), uric acid in saliva in the healthy population (*n* = 2), horses with ESGD+EGGD (*n* = 1), and uric acid in serum in the healthy population (*n* = 4), ESGD (n = 1), EGGD (*n* = 2), ESGD+EGGD (*n* = 1), and CIE (*n* = 2 and *n* = 1) populations, respectively. In these cases, the LLOQ was used for statistical purposes.

### 3.3. Adenosine Deaminase Results

Horses with EGGD lesions had higher salivary levels of ADAt (*p* = 0.009, β error = 0.02), ADA1 (*p* = 0.01, β error = 0.03), and ADA 2 (*p* = 0.003, β error = 0.11) than the healthy horses (Table 4).

Horses with CIE had higher salivary levels of ADA2 than the healthy population (*p* = 0.01, β error = 0.11). The non-survivors in the CIE population had higher levels in saliva of ADA1 (*p* = 0.04, β error = 0.47) and ADA2 (*p* = 0.002, β error = 0.20) than the healthy horses.

### 3.4. Changes in the Oxidative Stress Biomarkers in Saliva and Serum between the Healthy and Diseased Populations

**Figure 1:**
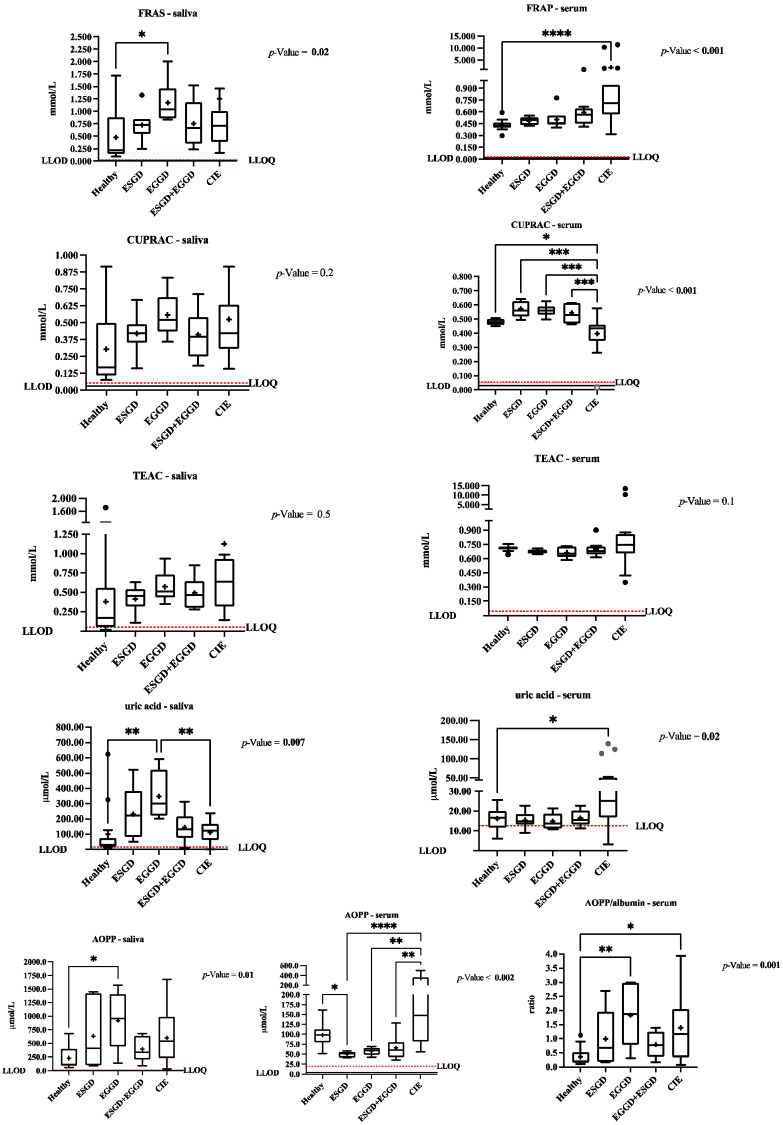
The ferric reducing ability of saliva/serum (FRAS/FRAP), cupric reducing antioxidant capacity (CUPRAC), Trolox equivalent antioxidant capacity (TEAC), uric acid, and advanced oxidation protein products (AOPP) and ratio AOPP/albumin results in healthy horses (*n* = 14), horses suffering Equine Squamous Gastric Disease (ESGD, *n* = 6), Equine Glandular Gastric Disease (EGGD, *n* = 8), ESGD + EGGD (*n* = 9), and colic of intestinal aetiology (CIE, *n* = 22). The plot shows the median (line within box), mean (the cross inside the box), 25th–75th percentiles (box), 5th and 95th percentiles (whiskers), and outliers (•). The dotted and continuous lines show the lower limit of detection (LLOD) and the lower limit of quantification (LLOQ). Asterisks indicate statistically significant differences between groups (* *p* <0.05, ** *p* <0.01; *** *p* <0.001; **** *p* <0.0001). The line under the asterisks indicates the groups that showed significant differences.

The values of the salivary and serum biomarkers evaluated in this study in the healthy and the diseased populations appear in Figure 1.

The levels of FRAS (*p* = 0.02, β error = 0.19), uric acid (*p* = 0.02, β error = 0.20), and AOPP (*p* = 0.01, β error = 0.14) in saliva, and the ratio AOPP/albumin (*p* = 0.009, β error = 0.13) in serum were higher in the horses with EGGD than in the healthy group.

In serum, horses with CIE showed increased levels of FRAP (*p* < 0.001; β error = 0.50), uric acid (*p* = 0.04; β error = 0.16), and the AOPP/albumin ratio (*p* = 0.04, β error = 0.13) than in the healthy horses. The CUPRAC level in serum was lower (*p* = 0.04, β error = 0.01) in horses suffering from CIE than in the healthy group.

### 3.5. Differences in the Salivary and Serum Oxidative Stress Biomarkers depending on the Colic Outcome

**Figure 2:**
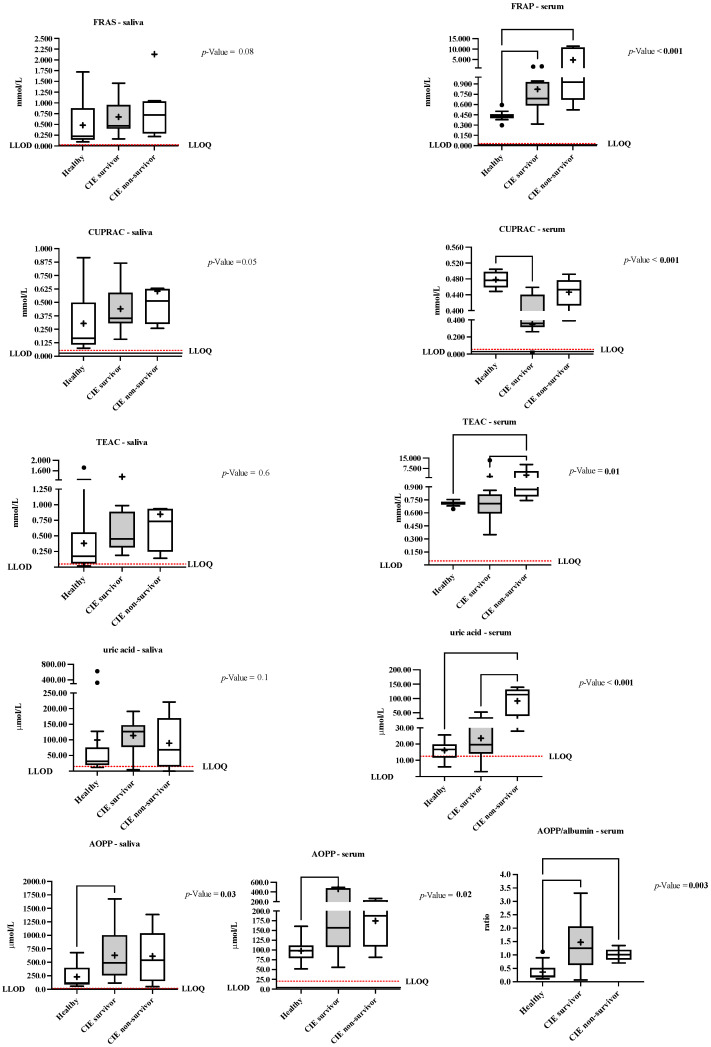
The ferric reducing ability of saliva/serum (FRAS/FRAP), cupric reducing antioxidant capacity (CUPRAC), Trolox equivalent antioxidant capacity (TEAC), uric acid, and advanced oxidation protein products (AOPP) and ratio AOPP/albumin results in healthy horses (*n* = 14, dark grey), and horses which survived to the CIE (*n* = 14, silver grey) vs. those that did not (non-survivors, *n* = 8, white). The plot shows the median (line within box), mean (the cross inside the box), 25th–75th percentiles (box), 5th and 95th percentiles (whiskers), and outliers (•). The dotted and continuous lines show the lower limit of detection (LLOD) and the lower limit of quantification (LLOQ).

In horses with CIE (Figure 2), both the survivors (*p* = 0.005 and *p* = 0.005) and non-survivors (*p* = 0.002 and *p* = 0.031) showed increased levels of FRAP (β error = 0.06) and AOPP/albumin ratio (β error = 0.04) compared with the healthy group. TEAC and uric acid levels in serum were higher (β error = 0.65 and β error = 0.01) in the non-survivor horses compared to both the healthy (*p* = 0.02 and *p* = 0.001) and survivors (*p* = 0.02 and *p* = 0.001) horses, respectively.

### 3.6. Associations between the Salivary and Serum Redox Profiles with the Inflammatory and Immunity Biomarkers

The correlations’ results are listed in Supplementary Table S2. Significant and high correlations (r = 0.70–0.89) in saliva were observed in the horses suffering from ESGD between FRAS (*p* = 0.014, *p* = 0.018, and *p* = 0.006), CUPRAC (*p* = 0.004, *p* = 0.007, and *p* = 0.005), and TEAC (*p* = 0.007, *p* = 0.009, and *p* = 0.018) with ADAt, ADA1, and ADA2 values, respectively, and in the horses suffering EGGD between FRAS (*p* < 0.001), CUPRAC (*p* = 0.001), and uric acid (*p* = 0.023) with ADA2. In serum, a high correlation in horses with ESGD was observed between TEAC and SAA levels (*p* = 0.003).

In the horses diagnosed with CIE, a moderate correlation (r = 0.58, *p* = 0.016) was observed between the SIRS score and the serum levels of uric acid.

## 4. Discussion

This is the first report where biomarkers of oxidative stress are measured in the saliva of horses to the best of the authors’ knowledge. The selected assays that integrated the oxidative stress profile used in our study showed an acceptable intra-assay imprecision, always below 10% (the limit recommended for automated methods is set at 15%) [30]. They also showed high correlation coefficients (r = 0.985 ± 0.018) and good linearity (slopes were not significantly different from one, and the intercepts were not zero) in serially diluted pools samples. These results demonstrate that these analytes can be measured in saliva and serum from horses, with the advantages of automation. However, these assays can be adapted to other formats, such as microplate readers or single spectrophotometers. Although most analytes measured in saliva and serum showed higher values than the LLOQ of the assays in the assessed populations, in some cases, the values of TEAC in saliva and uric acid in saliva and serum were below their LLOQ. Therefore, developing more sensitive assays would be valuable to detect those low values observed in some horse samples.

Changes in biomarkers of oxidative stress were observed in horses with EGGD and CIE. Horses suffering from EGGD showed increased values of the oxidant biomarker AOPP and the antioxidant biomarkers FRAS and uric acid in saliva, whereas changes were not observed in horses suffering from ESGD. The pathophysiology of ESGD has been related to factors that increase the exposure of the squamous mucosa to acid, whereas the EGGD pathophysiology has not been totally elucidated [10]. It is hypothesised that a breakdown of the normal defence mechanisms that protect the mucosa from acidic gastric contents from which it is accustomed could be the reason. In this sense, the increase in AOPP in saliva in horses with EGGD would indicate the involvement of oxidative stress in the pathogenesis of this disease, which is in line with the increase in malondialdehyde, an oxidant biomarker of lipid peroxidation detected in serum of horses with EGUS [9]. Increases in the ratio AOPP/albumin in serum were also found in the horses with EGGD in our study, while AOPP measured alone in serum did not show an increase. Therefore, the ratio of AOPP/albumin in the blood seems to detect the oxidant status better, as previously recommended [31], being highly correlated with the AOPP concentration in saliva in our study (data now shown). Possibly, the use of this ratio would not be needed in saliva due to the low amounts of albumin in that fluid. Therefore, according to our results, it could be hypothesised that after damage at a gastric mucosa level, there is an increase in the production of oxidative compounds that can be detected by the increase in the ratio of AOPP/albumin in serum and AOPP concentration in saliva. Then, the increase in the antioxidant activity in saliva could occur to compensate for this oxidative damage by the releasing of antioxidants components into saliva [16].

The fact that only horses with EGGD showed significant increases in the saliva of ADA and its isoenzymes, which are biomarkers of the immune system activation, could indicate an involvement of the immune system in EGGD. This would be in line with recent reports that relate an immune-mediated involvement in the pathophysiology of gastroduodenal disease, ulcerogenesis, and the healing process in horses [4,9,11,20]. ADA plays a role in the B and T lymphocytes’ differentiation and maturation from monocyte to macrophage [32], and since glandular mucosa inflammation in EGGD is associated with lymphoplasmacytic infiltration [11], this could be the reason for the high activity of this enzyme in this condition. However, no coincident changes in ADA from saliva and serum were observed, as has previously been reported in dogs [33] and horses [4], probably owing to a local releasing of this enzyme in the salivary gland independent of the ADA concentration in the blood, as also happens with other enzymes as the salivary alpha-amylase [34]. Moreover, some antioxidants in saliva, such as FRAS, CUPRAC, and uric acid, correlated with the ADA activity. This would indicate a relationship between the biomarkers of redox status and immune system and, therefore, the variations in oxidative status biomarkers in EGGD could be influenced by the changes in the immune system occurring in this disease and possibly to the lymphoplasmacytic infiltration [16].

In horses with CIE, the oxidative status changes were reflected in the serum, with changes in the antioxidant biomarkers FRAP, CUPRAC, and uric acid, and in the oxidative biomarker AOPP. The ratio of AOPP/albumin increasing in horses with CIE could reflect a disturbance in the oxidative status as previously described in horses with impactions [35]. Therefore, the damage to intestinal mucosa levels due to ischemic injury would only produce antioxidant changes reflected in the serum [35], but no antioxidants released into saliva. However, this hypothesis should be further corroborated in future studies. In addition, TEAC and uric acid levels in serum were higher in non-surviving horses than in survivors, but only serum uric acid showed a moderate correlation with the SIRS score. Uric acid in excess does not act as an antioxidant but induces cytokine and chemokine production, leading to activation of the inflammatory cascade, which may eventually cause endothelial dysfunction and fibrosis. Therefore, in humans, uric acid has been suggested to be a measure of sepsis state and progression [36]. A low correlation between the inflammatory status (SAA levels) and the salivary TEAC levels in horses with CIE was observed, but SAA was not correlated with TEAC in serum. Salivary ADA2 was higher in the non-survivor CIE group, thus corroborating results previously reported with CIE [4].

This report has some limitations and, therefore, should be considered as a pilot study. It is important to point out that the possible stressful situation of being in a new place could influence our results, mainly in the CIE group since they were sampled immediately after their arrival. Additionally, a gastroscopy was not performed in horses from the CIE group. Therefore, possible gastric ulceration could not be ruled out in these horses. In addition, although most of the analytes showed adequate statistical power, these results should be considered preliminaries. Therefore, further studies with a larger population of horses with ESGD, EGGD, and CIE with a stricter inclusion criterion to separate out their differences and causes should be performed to confirm our findings and to evaluate the ability of the analytes of this report for its clinical use, not only in the diagnosis but also to evaluate disease progression and monitoring of treatment. In addition, further studies should be performed to evaluate whether these oxidative status changes in horse saliva and serum are only observed in gastrointestinal diseases or other types of diseases not related to the gastrointestinal tract.

## 5. Conclusions

Based on this study, a panel of analytes related to oxidative status (FRAS/FRAP, CUPRAC, TEAC, uric acid, and AOPP) can be measured with sufficient reliability and sensitivity in both saliva and serum from horses using automated assays, being the first study that analytically validates them in horse saliva to the best of the authors’ knowledge. In addition, changes in the saliva FRAS, uric acid, and AOPP levels and serum ratio of AOPP/albumin from horses with gastric ulcers in the glandular mucosa, and in the serum FRAP, CUPRAC, uric acid levels, and the ratio of AOPP/albumin from horses suffering from CIE, were observed. These redox biomarkers’ changes in horses suffering from EGGD are correlated with the ADA activity in saliva, a biomarker related to the immune system. Overall, this report indicates, for the first time, that analytes related to redox status can be measured in the saliva of horses and can have potential use as biomarkers in this species. The evaluation of these analytes has advantages associated with the use of saliva, such as easy, non-painful, and non-invasive collection, and the possibility to be obtained in field conditions, with repeated sampling, producing minimum discomfort and anxiety.

## Figures and Tables

**Table 1:** Intra-assay coefficients of variation (CVs) in saliva and serum pool specimens with different ferric reducing abilities of saliva/serum (FRAS), cupric reducing antioxidant capacity (CUPRAC), Trolox equivalent antioxidant capacity (TEAC), uric acid, and advanced oxidation protein products (AOPP) levels.

Analytes	Pool Specimens	Saliva			Serum		
		Mean Values	CV (%)	Mean CV (SD)	Mean Value	CV (%)	Mean CV (SD)
FRAS/FRAP (mmol/L)	High	1.639	0.45	0.48 (0.14)	0.884	0.58	0.51 (0.13)
	Medium	0.439	0.36		0.427	0.36	
	Low	0.243	0.63		0.360	0.59	
CUPRAC (mmol/L)	High	0.867	0.54	0.81 (0.28)	0.878	0.98	1.26 (0.49)
	Medium	0.446	0.80		0.402	1.82	
	Low	0.256	1.10		0.229	0.97	
TEAC (mmol/L)	High	0.918	1.28	2.16 (1.67)	0.881	0.46	0.82 (0.54)
	Medium	0.613	1.11		0.742	1.44	
	Low	0.105	4.08		0.670	0.56	
uric acid (μmol/L)	High	515.69	0.35	1.33 (0.85)	21.41	4.30	7.84 (3.62)
	Medium	93.38	1.67		13.68	11.53	
	Low	75.54	1.95		13.09	7.69	
AOPP (μmol/L)	High	844.2	6.68	4.17 (2.72)	141.7	4.69	3.66 (0.95)
	Medium	112.2	1.27		78.6	2.81	
	Low	90.2	4.55		59.2	3.48	

**Table 2:** Linear regression analysis between expected and observed results from a linearity of dilution study of two pool specimens of horse saliva and serum (1 and 2) with different ferric reducing ability of saliva/serum (FRAS/FRAP), cupric reducing antioxidant capacity (CUPRAC), Trolox equivalent antioxidant capacity (TEAC), uric acid, and advanced oxidation protein products (AOPP) levels.

Analytes	Sample	Pool Specimens	Analyte Levels	Slope (95% CI)	Y-Intercept (95% CI)	*p*-Value	R^2^
FRAS/FRAP (mmol/L)	Saliva	1	1.644	0.92 (0.47–1.34)	0.17 (−0.22–0.57)	0.01	0.976
		2	0.438	1.03 (0.30–1.76)	−0.05 (−0.25–1.59)	0.03	0.949
	Serum	1	0.877	1.04 (0.61–1.47)	−0.07 (−0.30–0.16)	0.009	0.982
		2	0.426	1.05 (0.55–1.56)	−0.04 (−0.18–0.09)	0.01	0.976
CUPRAC (mmol/L)	Saliva	1	0.878	1.11 (0.77–1.45)	−0.12 (−0.30–0.07)	0.005	0.990
		2	0.402	1.23 (1.00–1.45)	−0.10 (−0.16–0.04)	0.002	0.996
	Serum	1	0.426	1.28 (0.40–2.15)	−0.15 (−0.41–0.11)	0.04	0.952
		2	0.446	1.31 (0.43–2.19)	−0.17 (−0.44–0.10)	0.05	0.954
TEAC (mmol/L)	Saliva	1	0.918	0.96 (0.92–1.01)	0.03 (0.01–0.06)	<0.001	1.000
		2	0.613	0.95 (0.92–0.99)	0.03 (0.02–0.04)	<0.001	1.000
	Serum	1	0.879	0.99 (0.73–1.25)	−0.01 (−0.15–0.13)	0.004	0.993
		2	0.742	0.96 (0.90–1.02)	0.03 (0.00–0.05)	<0.001	1.000
uric acid (μmol/L)	Saliva	1	517.48	0.99 (0.95–1.04)	0.09 (−0.14–0.31)	<0.001	1.000
		2	91.00	1.02 (0.90–1.14)	−0.02 (−0.12–0.09)	<0.001	0.999
	Serum	1	21.41	1.09 (0.76–1.42)	−0.04 (−0.11–0.04)	0.005	0.990
		2	14.28	1.05 (0.75–1.34)	−0.04 (−0.05–0.03)	0.004	0.992
AOPP (μmol/L)	Saliva	1	630.0	0.97 (0.66–1.29)	−1.63 (−120.3–117.0)	0.006	0.989
		2	112.2	1.00 (0.40–1.60)	−7.08 (−49.1–35.0)	0.02	0.963
	Serum	1	141.7	0.99 (0.99–1.00)	0.17 (−0.17–0.52)	<0.001	1.000
		2	78.6	0.99 (0.93–1.05)	0.50 (−2.43–3.43)	<0.001	1.000

CI = confidence interval.

**Table 3:** The low limit of detection (LLOD) and low limit of quantification (LLOQ) in horse saliva and serum obtained for the ferric reducing ability of saliva/serum (FRAS/FRAP), cupric reducing antioxidant capacity (CUPRAC), Trolox equivalent antioxidant capacity (TEAC), uric acid, and advanced oxidation protein products (AOPP) assays.

Parameters	Samples Types	FRAP	CUPRAC	AHH	Uric Acid	AOPP
		mmol/L	mmol/L	mmol/L	µmol/L	µmol/L
LLOQ	SALIVA	<0.025	<0.054	0.049	<14.87	<12.7
	SERUM	<0.029	<0.056	0.046	12.49	20.2
LLOD		0.015	0.029	0.000	0.00	3.8

**Table 4:** Means and 95% confidence interval (CI) or medians and interquartile range [IQR] results of the total adenosine deaminase (ADAt) and isoenzymes (ADA1 and ADA2) in saliva and serum in a population of healthy horses (*n* = 14), horses suffering Equine Squamous Gastric Disease (ESGD, *n* = 6), Equine Glandular Gastric Disease (EGGD, *n* = 8), ESGD+EGGD (*n* = 9), and horses with colic of intestinal aetiology (CIE, *n* = 22) which survived to the CIE (*n* = 14) vs. those that did not (non-survivors, *n* = 8).

Horse Population	ADAt Serum (IU/L)	ADAt Saliva (IU/L)	ADA1 Saliva (IU/L)	ADA2 Saliva (IU/L)
Healthy horses	0.30 [0.20–0.40]	38.95 [15.58–98.75]	38.27 [14.72–108.30]	0.50 [0.30–1.45]
ESGD	0.60 [0.40–0.60]	397.60 (224.50–570.70)	391.50 (165.50–616.60)	5.74 (1.25–10.23)
EGGD	0.45 [0.38–0.68]	672.50 (206.80–1138.00) ^1^	662.20 (202.60–1122.00) ^1^	10.32 (3.80–16.84) ^1^
ESGD + EGGD	0.40 [0.30–0.60]	623.90 (228.50–1019.00) ^1^	629.20 (188.20–1070.00) ^1^	11.09 (3.20–18.98) ^1^
CIE	0.30 [0.20–0.30]	143.00 [42.60–513.30]	138.00 [39.85–505.00]	5.70 [2.48–8.25] ^1^
survivors	0.20 [0.10–0.30]	85.10 [12.50–544.60]	86.45 [14.68–298.70]	5.35 [1.38–7.90] ^2^
non-survivors	0.30 [0.30–0.40]	179.20 [108.30–550.10]	184.00 [110.60–572.80] ^2^	4.85 [3.05–16.08] ^2^

^1^ Significant differences from horses suffering ESGD, EGGD, ESGD + EGGD, and CIE with the healthy horses: ADAt saliva, *p* < 0.01 with the EGGD, and *p* < 0.01 with the ESGD + EGGD. ADA1 saliva, *p* < 0.05 with the EGGD, *p* < 0.05 with the ESGD + EGGD. ADA2 saliva, *p* < 0.01 with the EGGD, *p* < 0.01 with the ESGD + EGGD, *p* < 0.01 with the CIE. ^2^ Significant differences from horses which survived to the CIE (survivors) and those that did not (non-survivors) with the healthy horses: ADA1 saliva, *p* < 0.05 with the non-survivor horses with CIE. ADA2 saliva, *p* < 0.05 with the survivor horses with CIE, and *p* < 0.01 with the non-survivor horses with CIE.

## Data Availability

Not applicable.

## Supplementary Materials

The following supporting information can be downloaded at: https://www.mdpi.com/article/10.3390/ani12050667/s1, Table S1: Overview of horses included in the healthy and diseased groups suffering from Equine Gastric Ulcer Syndrome (EGUS, stratified into Equine Squamous Gastric Disease (ESGD), Equine Glandular Gastric Disease (EGGD), or both (ESGD + EGGD)), and colic of intestinal aetiology (CIE). The information about the systemic inflammatory response syndrome (SIRS) score is also shown if appropriate; Table S2: Spearman correlation coefficients (r) and 95% confidence intervals (CI) in horses suffering from Equine Squamous Gastric Disease (ESGD, *n* = 15) and/or Equine Glandular Gastric Disease (EGGD, *n* = 17), and colic of intestinal aetiology (CIE) between the salivary and serum redox profile (the ferric reducing ability of saliva/serum—FRAS/FRAP-, cupric reducing antioxidant capacity—CUPRAC-, Trolox equivalent antioxidant capacity—TEAC-, uric acid, and the advanced oxidation protein products—AOPP-) with the serum amyloid A (SAA) and salivary and serum total adenosine deaminase (ADAt) and its salivary isoenzymes (ADA1 and ADA2), and the systemic inflammatory response syndrome (SIRS) score in the CIE horses.
