# Supplementary material for: Changes in Oxidative Status Biomarkers in Saliva and Serum in the Equine Gastric Ulcer Syndrome and Colic of Intestinal Aetiology: A Pilot Study
Source: Animals (Basel). 2022 Mar 7;12(5):667. doi: 10.3390/ani12050667 (PMC8909870; doi:10.3390/ani12050667)
Supplement: Supplementary file 1 [file animals-12-00667-s001.zip › Supplementary Table S1.pdf]

Diseased population

Age (years)	Gender	Breed	Diagnosis	SIRS score	Outcome
11	Mare	Warmblood breed	EGGD	0	survivor
11	Mare	Warmblood breed	ESGD+EGGD	0	survivor
10	Gelding	Warmblood breed	EGGD	0	survivor
15	Gelding	Warmblood breed	ESGD+EGGD	0	survivor
13	Mare	Warmblood breed	ESGD+EGGD	0	survivor
8	Gelding	Warmblood breed	ESGD	0	survivor
5	Gelding	Warmblood breed	ESGD+EGGD	0	survivor
15	Gelding	Warmblood breed	EGGD	0	survivor
9	Mare	Warmblood breed	EGGD	0	survivor
14	Mare	Crossbred	ESGD+EGGD	0	survivor
10	Gelding	Pony	EGGD	0	survivor
17	Gelding	Warmblood breed	ESGD	0	survivor
11	Gelding	Warmblood breed	EGGD	0	survivor
6	Mare	Trotter breed	ESGD+EGGD	0	survivor
13	Gelding	Friesian horse	ESGD+EGGD	0	survivor
14	Gelding	Warmblood breed	EGGD	0	survivor
13	Gelding	Warmblood breed	ESGD	0	survivor
5	Gelding	Quarter horse	ESGD	0	survivor
10	Mare	Warmblood breed	ESGD+EGGD	0	survivor
7	Mare	Pony	ESGD+EGGD	0	survivor
16	Stallion	Crossbred	ESGD	0	survivor
10	Mare	Pony	EGGD	0	survivor
7	Stallion	Pure Spanish horse	ESGD	1	survivor
6	Stallion	Pure Spanish horse	Proximal enteritis	2	survivor
16	Gelding	Warmblood breed	Large colon displacement	0	survivor
12	Gelding	Holsteiner	Large colon displacement	1	survivor
12	Gelding	Warmblood breed	Impaction of the jejune	2	survivor
11	Stallion	Pure Spanish horse	Impaction of Caecum	1	survivor
16	Gelding	Icelandic horse	Medical colic	1	survivor
4	Mare	Crossbreed	Impaction of the pelvic flexure	2	survivor
7	Gelding	Friesian horse	Impaction of the jejune	2	non-survivor
8	Stallion	Lusitanian horse	Large colon displacement	2	survivor
14	Mare	Crossbreed	Strangulating lesion in the small intestine	3	non-survivor
9	Gelding	Thoroughbred	Large colon displacement	0	survivor
4	Stallion	Arabian	Colitis	2	non-survivor
10	Stallion	Pure Spanish horse	Proximal enteritis	2	survivor
10	Mare	Arabian	Strangulating lesion in the small intestine	2	non-survivor
18	Gelding	Warmblood breed	Colitis	0	survivor
14	Gelding	Crossbreed	Nephrosplenic entrapment	3	non-survivor
18	Gelding	Crossbreed	Volvulus in the large colon	3	non-survivor
20	Mare	Crossbreed	Impaction of ileum and colitis	2	non-survivor
15	Gelding	Lusitanian horse	Impaction in the small colon	2	non-survivor
5	Mare	Lusitanian horse	Impaction of the pelvic flexure	0	survivor
17	Mare	Warmblood breed	Impaction of the pelvic flexure	1	survivor
12	Mare	Crossbreed	Proximal enteritis	3	survivor

Healthy population

Age (years)	Gender	Breed	Diagnosis	SIRS score	Outcome
4	Mare	Warmblood breed	N/A	0	N/A
20	Mare	Pony	N/A	0	N/A
18	Mare	Crossbreed	N/A	0	N/A
14	Mare	Knabstrupper	N/A	0	N/A
4	Stallion	Pure Spanish horse	N/A	0	N/A
12	Stallion	Arabian	N/A	0	N/A
4	Gelding	Crossbreed	N/A	0	N/A
5	Gelding	Warmblood breed	N/A	0	N/A
8	Gelding	Pure Spanish horse	N/A	0	N/A
14	Gelding	Pony	N/A	0	N/A
20	Mare	Pony	N/A	0	N/A
17	Gelding	Crossbreed	N/A	0	N/A
10	Gelding	Pure Spanish horse	N/A	0	N/A
8	Mare	Crossbreed	N/A	0	N/A
